# Potent CCR3 Receptor Antagonist, SB328437, Suppresses Colonic Eosinophil Chemotaxis and Inflammation in the *Winnie* Murine Model of Spontaneous Chronic Colitis

**DOI:** 10.3390/ijms23147780

**Published:** 2022-07-14

**Authors:** Rhiannon T. Filippone, Narges Dargahi, Rajaraman Eri, Jose A. Uranga, Joel C. Bornstein, Vasso Apostolopoulos, Kulmira Nurgali

**Affiliations:** 1 Institute for Health and Sport, Victoria University, Western Centre for Health Research and Education, Sunshine Hospital, Melbourne, VIC 3021, Australia; rhiannon.filippone@live.vu.edu.au (R.T.F.); narges.dargahi@vu.edu.au (N.D.); kulmira.nurgali@vu.edu.au (K.N.); 2 School of Health Sciences, The University of Tasmania, Launceston, TAS 7248, Australia; rajaraman.eri@utas.edu.au; 3 Department of Basic Health Sciences, University Rey Juan Carlos (URJC), 28922 Alcorcón, Spain; jose.uranga@urjc.es; 4 High Performance Research Group in Physiopathology and Pharmacology of the Digestive System (NeuGut), University Rey Juan Carlos (URJC), 28922 Alcorcón, Spain; 5 Department of Anatomy and Physiology, The University of Melbourne, Melbourne, VIC 3010, Australia; j.bornstein@unimelb.edu.au; 6 Immunology Program, Australian Institute of Musculoskeletal Science (AIMSS), Melbourne, VIC 3021, Australia; 7 Department of Medicine-Western Health, Faculty of Medicine, Dentistry and Health Sciences, The University of Melbourne, Melbourne, VIC 3010, Australia; 8 Regenerative Medicine and Stem Cells Program, Australian Institute of Musculoskeletal Science (AIMSS), Melbourne, VIC 3021, Australia

**Keywords:** eosinophil accumulation, CCR3 receptor, SB328437, chronic intestinal inflammation, inflammatory bowel disease

## Abstract

Eosinophils and their regulatory molecules have been associated with chronic intestinal inflammation and gastrointestinal dysfunctions; eosinophil accumulation in the gut is prominent in inflammatory bowel disease (IBD). The chemokine receptor CCR3 plays a pivotal role in local and systemic recruitment and activation of eosinophils. In this study, we targeted CCR3-ligand interactions with a potent CCR3 receptor antagonist, SB328437, to alleviate eosinophil-associated immunological responses in the *Winnie* model of spontaneous chronic colitis. *Winnie* and C57BL/6 mice were treated with SB328437 or vehicle. Clinical and histopathological parameters of chronic colitis were assessed. Flow cytometry was performed to discern changes in colonic, splenic, circulatory, and bone marrow-derived leukocytes. Changes to the serum levels of eosinophil-associated chemokines and cytokines were measured using BioPlex. Inhibition of CCR3 receptors with SB328437 attenuated disease activity and gross morphological damage to the inflamed intestines and reduced eosinophils and their regulatory molecules in the inflamed colon and circulation. SB328437 had no effect on eosinophils and their progenitor cells in the spleen and bone marrow. This study demonstrates that targeting eosinophils via the CCR3 axis has anti-inflammatory effects in the inflamed intestine, and also contributes to understanding the role of eosinophils as potential end-point targets for IBD treatment.

## 1. Introduction

Inflammatory bowel disease (IBD) encompasses ulcerative colitis (UC) and Crohn’s disease (CD), which are both characterized by the infiltration of leukocytes into the inflamed colon and its structural damage [1,2,3]. Although the precise mechanism behind IBD pathogenesis remains unclear, accumulating evidence has indicated that leukocytes such as T-lymphocytes, eosinophils, neutrophils, and macrophages manifest in, and contribute to, the pathological lesions in the inflamed intestines of IBD patients [4,5]. Among these infiltrating leukocytes, eosinophils have been described as relentlessly infiltrating the inflamed intestines of both IBD patients and animal models of colitis [6,7,8,9]. Eosinophil accumulation in the inflamed intestines coincides with chronic intestinal inflammation, disease severity, and impediments to gastrointestinal (GI) functions during colitis [10,11]. It has been postulated that eosinophils can interact with nerve fibers innervating the GI tract, as previously observed in patients with IBD and acute models of colitis [12,13]. In the intestinal mucosa, eosinophils are functionally required to regulate intestinal integrity and provide provisional immunity against antigen invasion. However, changes to eosinophil-associated regulatory molecules can elicit eosinophil activation, by enhancing pro-inflammatory immunological cascades during intestinal inflammation [7,14].

Eosinophils are pro-inflammatory leukocytes that predominately originate from hematopoietic stem cell (HPSC) precursors in the bone marrow [15,16]. HPSCs are influenced by several transcription factors [17] and cytokines, such as interleukin (IL)-5, IL-3, and granulocytes monocyte-colony stimulating factor (GM-CSF), to commit to eosinophil progenitor cells (EPoCs) [18,19]. Alterations to one or more of these EPoC-associated cytokines can implicate eosinophil development during disease activity [20,21]. Eosinophils have bilobed nuclei and can be characterized by their highly dense granule-enriched cytoplasm via flow cytometry side scatter (SSC)^HI^ and positive expression of cluster differentiation (CD)45 [22]. Subsequently, eosinophils can be isolated with the cysteine–cysteine chemokine receptor (CCR)-3 (CD193) and a combination of CD11b and/or Sialic acid-binding immunoglobulin-like lectin (Siglec) receptor family (Siglec-F for mice; Siglec-8 for humans) [22,23,24,25,26]. Eosinophils are vital for defensive roles against the viral and parasitic pathogens that typically reside in mucosal regions of the lungs, uterus, intestines, and thymus [27]. Abnormalities of EPoC-associated cytokines and receptor expression can affect the activation, maturation, and infiltration of eosinophils in the inflamed and infected tissue sites [7].

Infiltration of eosinophils to the sites of intestinal inflammation is typically mediated by a chemoattractant gradient [2,28,29]. Chemokines are small proteins (7–15 kDa) that interact with chemokine G-protein coupled receptors to orchestrate immunological responses and leukocyte infiltration within the GI tract [2,30]. Chemokines can be divided into four main subfamilies: C, CC, CXC, and CX3C, depending on the position of the first two cysteine residues [2]. Chemokines and their receptors cause selective leukocyte trafficking and their activation at the sites of GI inflammation [31,32]. CCR3 receptors interacting with chemokine ligands, including eotaxin-1 (CCL11), eotaxin-2 (CCL24), eotaxin-3 (CCL26), Regulated on Activation, Normal T Cell Expressed and Secreted (RANTES; CCL5), monocyte chemotactic protein-2 (MCP-2; CCL8), monocyte chemotactic protein-3 (MCP-3; CCL7), monocyte chemotactic protein-4 (MCP-4; CCL13), and Mucosae-Associated Epithelial Chemokine (MEC; CCL28), stimulate eosinophil migration, adhesion, and activation [33,34,35,36]. Elevated serum levels of CCL11 and CCL5 chemokines have been reported to correlate with eosinophil infiltration in IBD pathogenesis [36,37,38,39]. Eosinophil activation and trafficking are enhanced by a T-helper (Th)-2 lymphocyte-mediated immune response in the inflamed intestine [40]. Th-2 lymphocytes release and influence other cells to secrete CCL11 and CCL5 [29,41]. In addition, Th-2 cells release the appropriate cytokine milieu, including IL-5, GM-CSF, and IL-4, facilitating the activation of eosinophils [29,42,43,44]. Degranulation of activated eosinophils leads to the release of toxic positively-charged cationic proteins: eosinophil-derived neurotoxin (EDN), eosinophil cationic protein (ECP), eosinophil peroxidase (EPO), and major basic protein (MBP) found in cytoplasmic granules [45,46,47]. These cationic proteins typically play integral roles in host defense during parasitic assaults, but the levels of the eosinophil constituents increase in gut lavage during IBD pathogenesis [48,49]. Eosinophils exert protective attributes during intestinal inflammation [50]. Additionally, significant gross morphological damage, severe colitis, and incompetent biosynthesis of anti-inflammatory derivatives, such as protectin D1 (PD1), correlate with exaggerated pro-inflammatory cascades in vivo in the mouse model, DSS-induced colitis with depleted eosinophils [51]. These findings suggest the importance of eosinophils in facilitating the wound healing process during IBD pathogenesis [50,52]. On the contrary, studies have demonstrated that eosinophil by-products activate other immune cells [46], leading to host tissue toxicities [45,53,54]. It has been postulated that eosinophil activity in the inflamed intestine results in clinical symptoms and GI dysfunction [55,56,57,58]. Thus, therapies targeting eosinophil infiltration and activation have been considered [13,59,60,61].

Targeting eosinophil accumulation via CCR3-evoked chemotaxis has been proposed as a viable candidate for alleviating damage to the inflamed GI tract [1,7,13,62]. In the inflamed intestines from patients with IBD and corresponding animal models of colitis, persistent accumulation of eosinophils localizing with nerve fibers has been observed, resulting in altered GI functions [12,13,63,64]. Targeting CCR3-mediated eosinophil recruitment has been said to dampen intestinal inflammation, permeability, and dysfunction, as observed in chemical-induced, chemokine deficient, and the SAMP1/SkuSlc spontaneous models of intestinal inflammation [13,61,62]. Animal models of chemically-induced colitis using dextran sodium sulfate (DSS) and trinitrobenzene sulfonic acid (TNBS) are commonly used, as they are accessible, inexpensive, and quick to develop, but unfortunately lack a true representation of human IBD [65]. Therefore, models of spontaneous colitis are invaluable for developing novel therapeutic interventions, as they closely resemble IBD pathogenesis [65]. For example, the SAMP1/SkuSlc mouse model with lesions in the ileum closely represents active CD [65]. On the contrary, the *Winnie* murine model of spontaneous chronic colitis primarily affects the large bowel and closely resembles UC [66,67,68]. *Winnie* mice have a missense mutation of the *Muc2* mucin gene, causing endoplasmic reticulum stress to goblet cells, resulting in mucosal damage [69,70]. The *Muc2* mucin gene is responsible for maintaining the luminal gel-forming mucin layer, which is considered the first line of defense of the GI tract from intestinal microbiota [71]. In IBD patients, reduced levels or absence of *Muc2* gene expression and reduced Muc2 production and secretion lead to a thinner mucus layer, epithelial cell damage, increased intestinal permeability, and enhanced susceptibility to luminal toxins within the gut [71]. In *Winnie* mice, defects to the protective mucin layer increase intestinal susceptibility to antigens, resulting in an abrupt immunological response, microbiota dysbiosis, enteric neuropathy, and alterations in the normal functioning of the GI tract foreseen by changes to the milieu of intestinal cells in the inflamed colon similar to human IBD [67,68,69,70,72,73]. Spontaneous colitis develops in all *Winnie* mice by 6 weeks of age in pathogen-free conditions; it progresses over time and results in severe colitis by the age of 12–16 weeks. To date, no study has investigated eosinophils and their accumulating factors as potential therapeutic endpoints in the *Winnie* murine model of spontaneous chronic colitis.

The premise of this study was to determine the efficacy and anti-inflammatory properties of a highly selective and potent CCR3 receptor antagonist, SB328437 (SB3), by employing the *Winnie* mouse model of spontaneous chronic colitis. This is the first compressive study on the efficacy of an antagonist that specifically inhibits CCR3 receptors and partially blocks CCL11 on eosinophil activity. The changes to chemokines (CCL11 and CCL5) and cytokines (GM-CSF, IL-3, IL-4, IL-5) associated with the differentiation, maturation, activation, and trafficking of eosinophils to sites of inflammation were investigated.

## 2. Results

### 2.1. SB3 Treatments Improved Disease Activity in Winnie Mice

**Figure 1:**
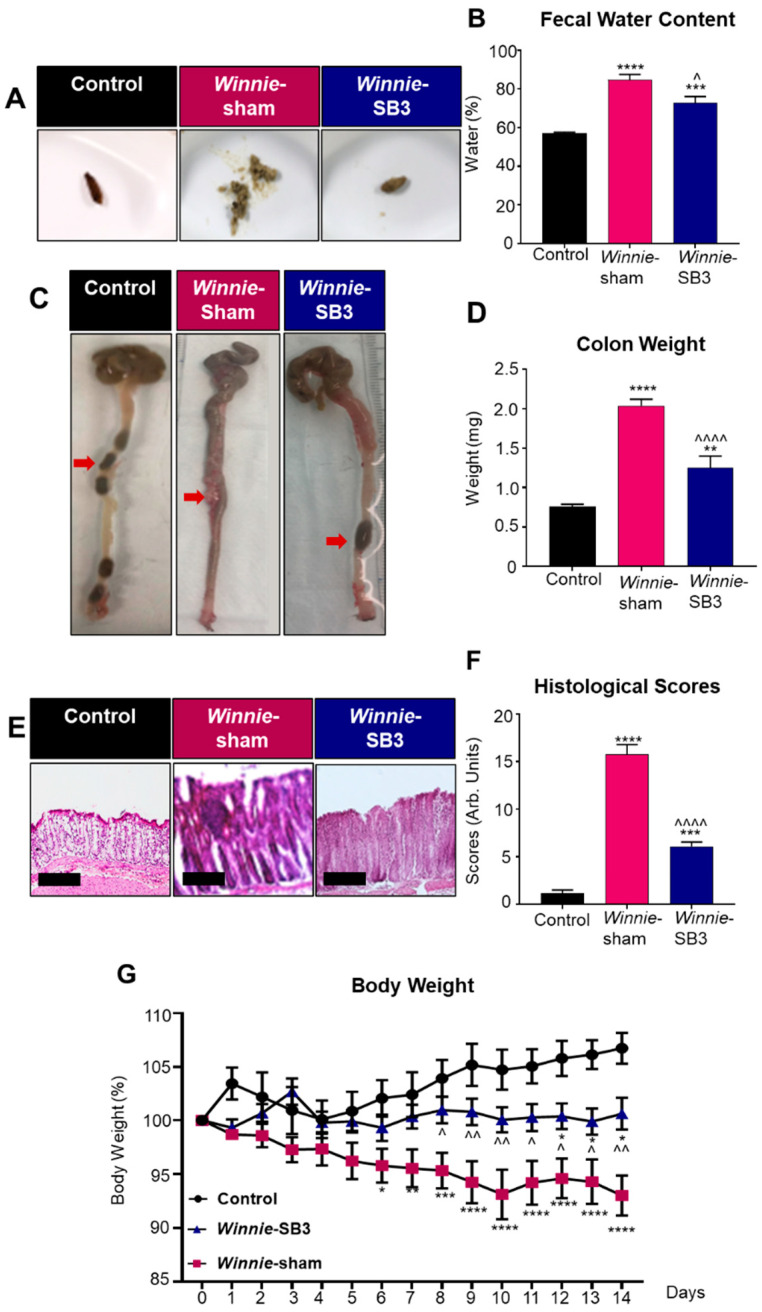
The effects of SB3 treatment on disease activity in *Winnie* mice. (**A**) Representative images of solid fresh fecal pellets from C57BL/6 healthy controls, loose and watery fecal masses from sham-treated *Winnie* mice, and improved fecal pellet formation after 14 days of SB3 treatment of *Winnie* mice. (**B**) The percentage of fecal water content (difference between wet and dry weight) was measured on day 14 of treatments (*n* = 6/group). (**C**) Images of colons showing soft contents in sham-treated *Winnie* mice compared to C57BL/6 controls and SB3-treated *Winnie* mice (red arrows). (**D**) Weights (mg) of the colons from C57BL/6 control (*n* = 8), sham-treated (*n* = 10), and SB3-treated (*n* = 8) *Winnie* mice were measured on day 15. (**E**) H&E-stained colon cross-sections from C57BL/6 control, sham-treated, and SB3-treated *Winnie* mice. Scale bar 20 µm. (**F**) Histological scores (arbitrary units) determined by quantification of gross morphological parameters in the colon (*n* = 7/group). (**G**) Measurements of body weights in C57BL/6 control (*n* = 11), sham-treated (*n* = 12), and SB3-treated (*n* = 11) *Winnie* mice over the 14-day treatment period. Data expressed as mean ± SEM, * *p* < 0.05, ** *p* < 0.01, *** *p* < 0.001, **** *p* < 0.0001 compared to C57BL/6 control mice; ^ *p* < 0.05, ^^ *p* < 0.01, ^^^^ *p* < 0.0001 compared to *Winnie*-sham treated mice.

Diarrhea is a prominent symptom that is typically observed in *Winnie* mice compared to solid fecal pellets in control animals (Figure 1A). Diarrhea was confirmed by measuring fecal water content in freshly collected feces. Higher levels of fecal water content (84.9 ± 2.7%, *n* = 6) were observed in feces from sham-treated *Winnie* mice on day 14 of treatment compared to C57BL/6 controls (57.0 ± 0.7%, *p* < 0.0001, *n* = 6; Figure 1B). In contrast, the fecal water content in SB3-treated *Winnie* mice was lower (72.9 ± 3.2%, *p* < 0.05, *n* = 6; Figure 1B) than in sham-treated *Winnie* mice but remained elevated when compared to controls (*p* < 0.001; Figure 1B). Weights of excised colons were determined on day 15 (Figure 1C,D). Colons from sham-treated *Winnie* mice were heavier (2.0 ± 0.1 mg, *p* < 0.0001, *n* = 10) and contained smooth fecal masses compared to the colons from control mice (0.7 ± 0.03 mg, *n* = 8; Figure 1C), which contained hard fecal pellets (Figure 1C). Reduced weights of colons and formation of harder pellets were observed in the colons from SB3-treated *Winnie* mice (1.3 ± 0.2 mg, *n* = 8; Figure 1C,D) when compared to sham-treated *Winnie* mice (*p* < 0.0001), although the former was not comparable to the weights of colons from C57BL/6 control mice (*p* < 0.01; Figure 1D). Gross morphological changes to epithelial crypts, leukocyte infiltration, ulcerations, and epithelial damage were evaluated in H&E stained colonic cross-sections in all experimental groups (Figure 1E). Sham-treated *Winnie* mice had significantly higher histological scores (15.8 ± 1.0 arb. units, *n* = 7) than C57BL/6 controls (1.2 ± 0.3 arb. units, *p* < 0.0001, *n* = 7; Figure 1F). The SB3 treatment dampened histological scores in *Winnie* mice (6.1 ± 0.4 arb. units, *n* = 7; *p* < 0.0001 compared to sham-treated *Winnie* mice), but not to the control levels (*p* < 0.0001; Figure 1F). The body weights of C57BL/6 control, *Winnie* sham-treated, and *Winnie* SB3-treated mice were recorded over the 14-day treatment period (Figure 1G). Body weights of sham-treated *Winnie* mice declined from day 6 to day 14 when compared to controls. Although the body weights of SB3-treated *Winnie* mice (*n* = 11) were higher than the body weights of sham-treated *Winnie* mice (*p* < 0.01, *n* = 12), they remained lower than those of C57BL/6 controls at day 14 (*p* < 0.05, *n* = 11; Figure 1G) (Table 1). No obvious adverse effects of the treatment were observed.

### 2.2. SB3 Treatment Reduced Eosinophil Accumulation in the Inflamed Colons of Winnie Mice

**Figure 2:**
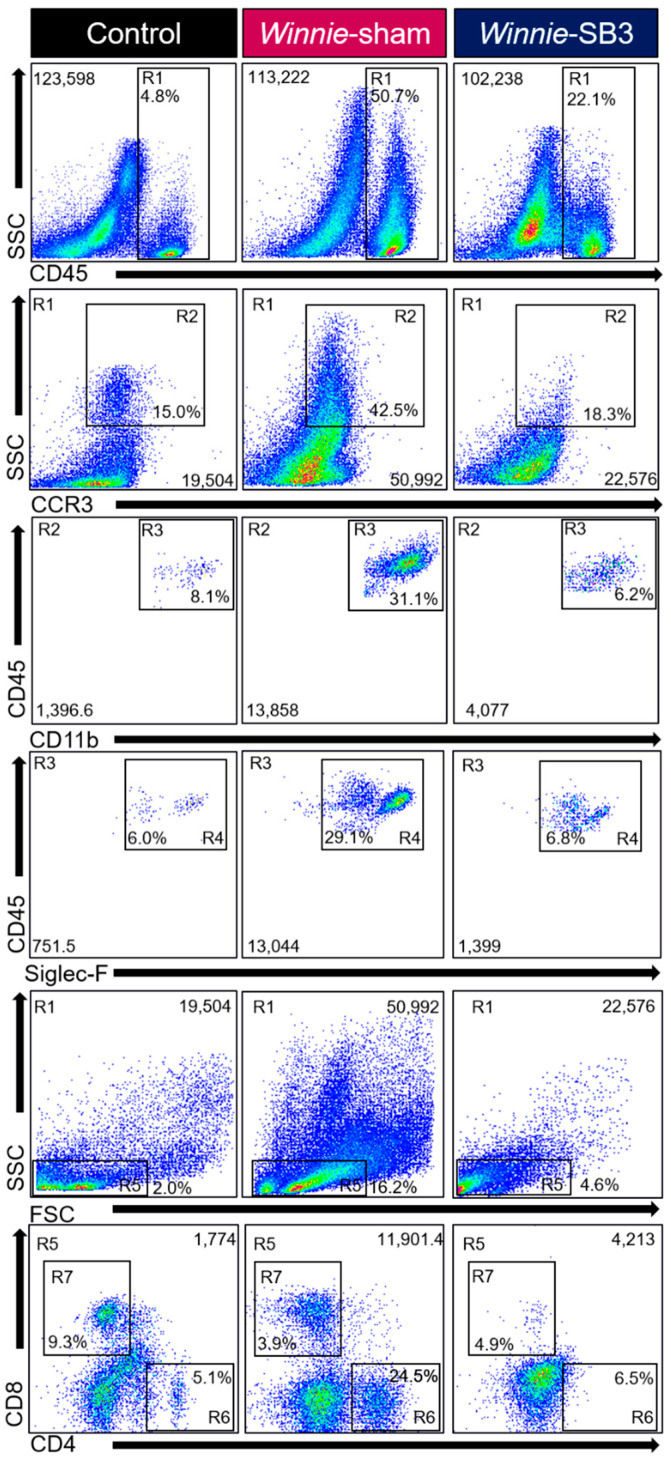
Gating strategy employed to characterize leukocytes, eosinophils, and T-lymphocytes in isolated single-cell suspensions from control, sham-treated, and SB3-treated *Winnie* animals. Region (R1) Viable cells were gated to isolate leukocytes based on SSC^LO/HI^ against CD45^+^ cells. Subsequent gating was based on (R2) SSC^HI^ and CCR3^+^, followed by (R3) CD11b^+^ and (R4) Siglec-F^+^ expressions to delineate eosinophil subsets. Based on (R1) viable CD45^+^ subsets, (R5) characterized SSC^LO^ and FSC^LO^ lymphocytes to isolate (R6) CD4^+^ T helper and (R7) CD8^+^ cytotoxic T cell subsets.

**Figure 3:**
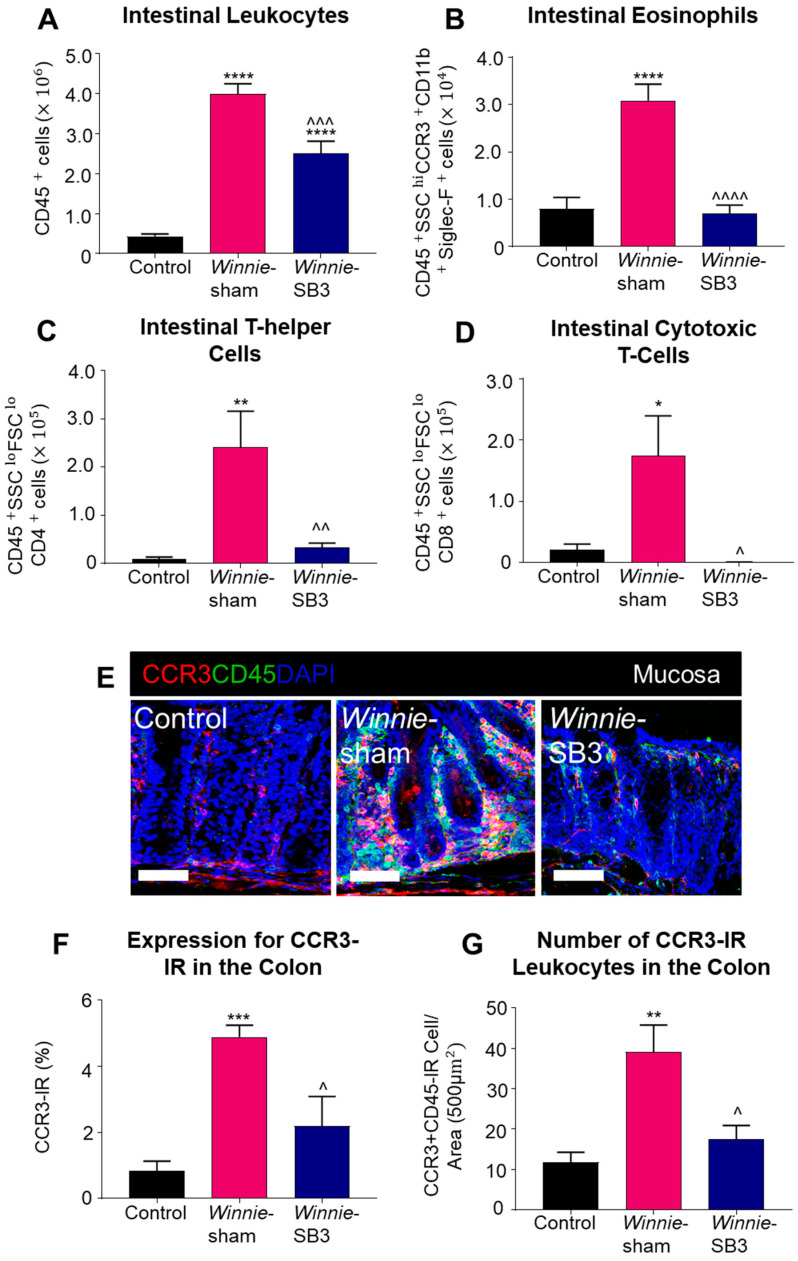
The effects of SB3 treatments on the CCR3-axis in the colons of *Winnie* mice. (**A**) Cell counts for CD45^+^ leukocytes quantified in controls, sham-treated *Winnie* mice, and SB3-treated *Winnie* mice (*n* = 8/group). The number of CD45^+^SSC^HI^CCR3^+^CD11b^+^Siglec-F^+^ eosinophils (**B**), CD45^+^SSC^LO^FSC^LO^CD4^+^ T-helper cells (**C**), and CD45^+^SSC^LO^FSC^LO^CD8^+^ cytotoxic T cells (**D**) in the colons from C57BL/6 controls, sham-treated, and SB3-treated *Winnie* mice (*n* = 5/group). (**E**). Antibodies against CCR3 receptors (red), pan leukocyte marker CD45 (green), and nuclear marker DAPI (blue) discerned CD45^+^CCR3^+^DAPI^+^-IR cells in the mucosa of the distal colon cross-sections of C57BL/6 control, sham-treated, and SB3-treated *Winnie* mice. Scale bar 50 μm. (**F**) Percentage of CCR3 immunoreactivity within the intestinal mucosa of C57BL/6 control, sham-treated, and SB3-treated *Winnie* mice (*n* = 5/group). (**G**) Quantification of the number of CD45^+^CCR3^+^DAPI^+^-IR cells infiltrating the intestinal mucosa in C57BL/6 control, sham-treated, and SB3-treated *Winnie* animals (per 500 µm^2^ area, *n* = 5/group). Data expressed as mean ± SEM; * *p* < 0.05, ** *p* < 0.01, *** *p* < 0.001, **** *p* < 0.0001 compared to control mice; ^ *p* < 0.05, ^^ *p* < 0.01, ^^^ *p* < 0.001, ^^^^ *p* < 0.0001 compared to sham-treated *Winnie* mice.

Flow cytometry was used to discriminate infiltrating leukocytes (CD45^+^), eosinophils (CD45^+^SSC^HI^CCR3^+^CD11b^+^Siglec-F^+^), and T lymphocyte subsets (CD45^+^SSC^LO^FSC^LO^CD4^+^ T helper and CD45^+^SSC^LO^FSC^LO^CD8^+^ cytotoxic cells) in isolated single-cell suspensions of the colons from control, sham-treated *Winnie,* and SB3-treated *Winnie* mice (Figure 2). Sham-treated *Winnie* mice had increased counts of CD45^+^ leukocyte populations in the colons compared to control animals (*p* < 0.0001, Table 2; Figure 3A). CD45^+^ leukocyte cell counts were attenuated in SB3-treated *Winnie* mice compared to those that were sham-treated (*p* < 0.001) but were higher than in control animals (*p* < 0.0001). Cell counts for intestinal CD45^+^SSC^HI^CCR3^+^CD11b^+^Siglec-F^+^ eosinophils were significantly higher in the colons from sham-treated *Winnie* mice compared to controls (*p* < 0.0001, Table 2; Figure 3B). SB3 treatment dampened CD45^+^SSC^HI^CCR3^+^CD11b^+^Siglec-F^+^ eosinophil cell counts in the colons from *Winnie* mice, compared to sham-treated *Winnie* mice (*p* < 0.0001) and to the control levels. Since T lymphocytes express CCR3 receptors, the effects of SB3 treatment on T helper (CD45^+^SSC^LO^FSC^LO^CD4^+^) and cytotoxic T (CD45^+^SSC^LO^FSC^LO^CD8^+^) cell subsets in the colon were determined. Increased counts for CD45^+^SSC^LO^FSC^LO^CD4^+^ T cells (*p* < 0.01) and CD45^+^SSC^LO^FSC^LO^CD8^+^ T cells (*p* < 0.05) were observed in sham-treated *Winnie* mice compared to control controls (Table 2; Figure 3C,D). SB3 treatment dampened the number of CD45^+^SSC^LO^FSC^LO^CD4^+^ T cells (*p* < 0.01) and CD45^+^SSC^LO^FSC^LO^CD8^+^ T cells (*p* < 0.05) compared to sham-treated *Winnie* mice, and to levels comparable to control mice.

The CCR3 receptor expression was determined via immunofluorescence in colon cross-sections in all experimental groups (Figure 3E). The level of CCR3 immunoreactivity was increased in the mucosa of the colons from sham-treated *Winnie* mice (4.8 ± 0.4%, *n* = 5) when compared to control mice (0.8 ± 0.3%, *p* < 0.001, *n* = 5; Figure 3F). SB3 treatment reduced CCR3 immunoreactivity (2.2 ± 0.9%, *n* = 5) when compared to sham-treated *Winnie* mice (*p* < 0.05). A pan leukocyte marker, an anti-CD45 antibody, was used to quantify the number of CCR3^+^CD45^++^-immunoreactive (IR) leukocytes within the colonic mucosa. Copious numbers of CCR3^+^CD45^+^-IR leukocytes infiltrating the colonic mucosa were observed in sham-treated *Winnie* mice (39.2 ± 6.7 cells per 500 µm^2^ area, *n* = 5) compared to controls (11.8 ± 2.5 cells/area, *p <* 0.01, *n* = 5; Figure 3G). Treatment with SB3 reduced the infiltration of CCR3^+^CD45^+^-IR leukocytes in the colonic mucosa of *Winnie* mice (17.5 ± 3.4 cells/area, *n* = 5) when compared to sham-treated *Winnie* mice (*p* < 0.05).

### 2.3. SB3 Treatments Had no Effect on Spleen Weights and Eosinophils in Winnie Mice

**Figure 4:**
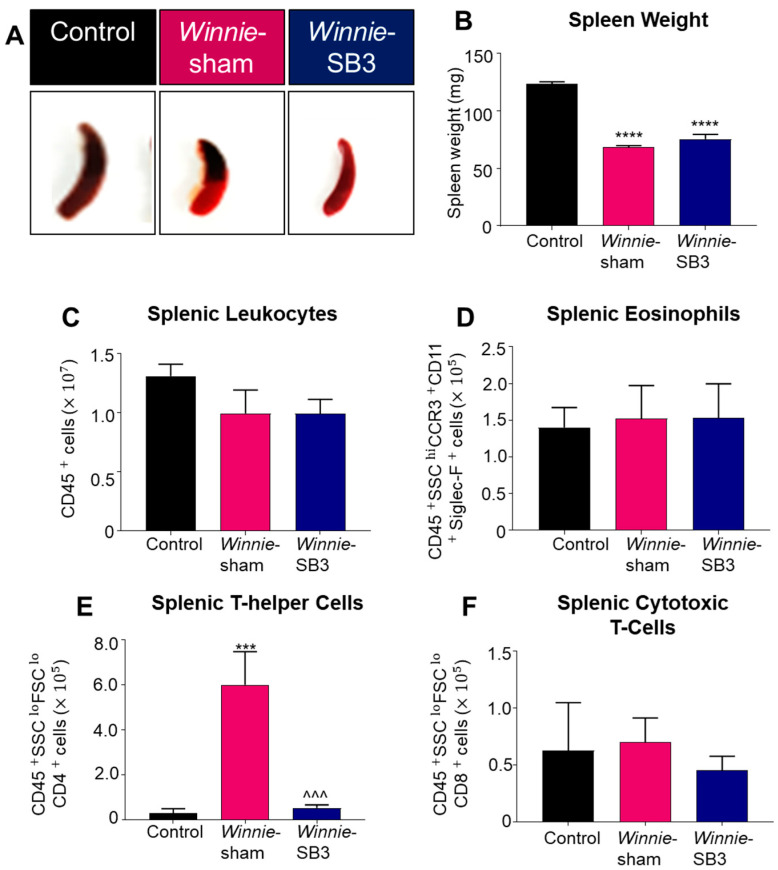
The effects of SB3 on spleen size and immune cell populations. (**A**) Representative images of spleens harvested from all experimental groups. (**B**) Weights (mg) of the spleens excised on day 15 from C57BL/6 control, sham-treated, and SB3-treated *Winnie* mice (*n* = 6/group). The number of CD45^+^ leukocytes (**C**), CD45^+^SSC^HI^CCR3^+^CD11b^+^Siglec-F^+^ eosinophils (**D**), CD45^+^SSC^LO^FSC^LO^CD4^+^ T helper lymphocytes (**E**), and CD45^+^SSC^LO^FSC^LO^CD8^+^ cytotoxic T lymphocytes (**F**) in the spleens from C57BL/6 control, sham-treated, and SB3-treated *Winnie* mice (*n* = 7/group). Data are expressed as mean ± SEM; *** *p* < 0.001, **** *p* < 0.0001 compared to control mice; ^^^ *p* < 0.001 compared to sham-treated *Winnie* mice.

Spleens were excised and weighed on day 15 from control, sham-treated *Winnie,* and SB3-treated *Winnie* mice (Figure 4A). Both sham-treated and SB3-treated *Winnie* mice had smaller spleens (68.4 ± 1.4 mg and 75.5 ± 4.1 mg, respectively, *n* = 6/group) than control mice (123.4 ± 2.0 mg, *p* < 0.0001, *n* = 6; Figure 4B). The total number of leukocytes and populations of eosinophils (CD45^+^SSC^HI^CCR3^+^CD11b^+^Siglec-F^+^), CD4^+^T cells (CD45^+^SSC^LO^FSC^LO^CD4^+^), and CD8^+^ T cells (CD45^+^SSC^LO^FSC^LO^CD8^+^) were counted in single-cell suspensions of spleens from experimental groups. No differences in the number of splenic CD45^+^ leukocytes, eosinophils, and CD8^+^ T cells were found between all experimental groups (Table 2; Figure 4C,D,F). However, a significant increase in CD4^+^ T cells in spleens from sham-treated *Winnie* mice (*p* < 0.001 compared to controls) was attenuated by SB3 treatment (*p* < 0.001 compared to sham-treated *Winnie* mice, Table 2; Figure 4E).

### 2.4. SB3 Treatments Attenuated Circulatory Eosinophils via Reducing Eosinophil-Associated Regulatory Molecules in Winnie Mice

**Figure 5:**
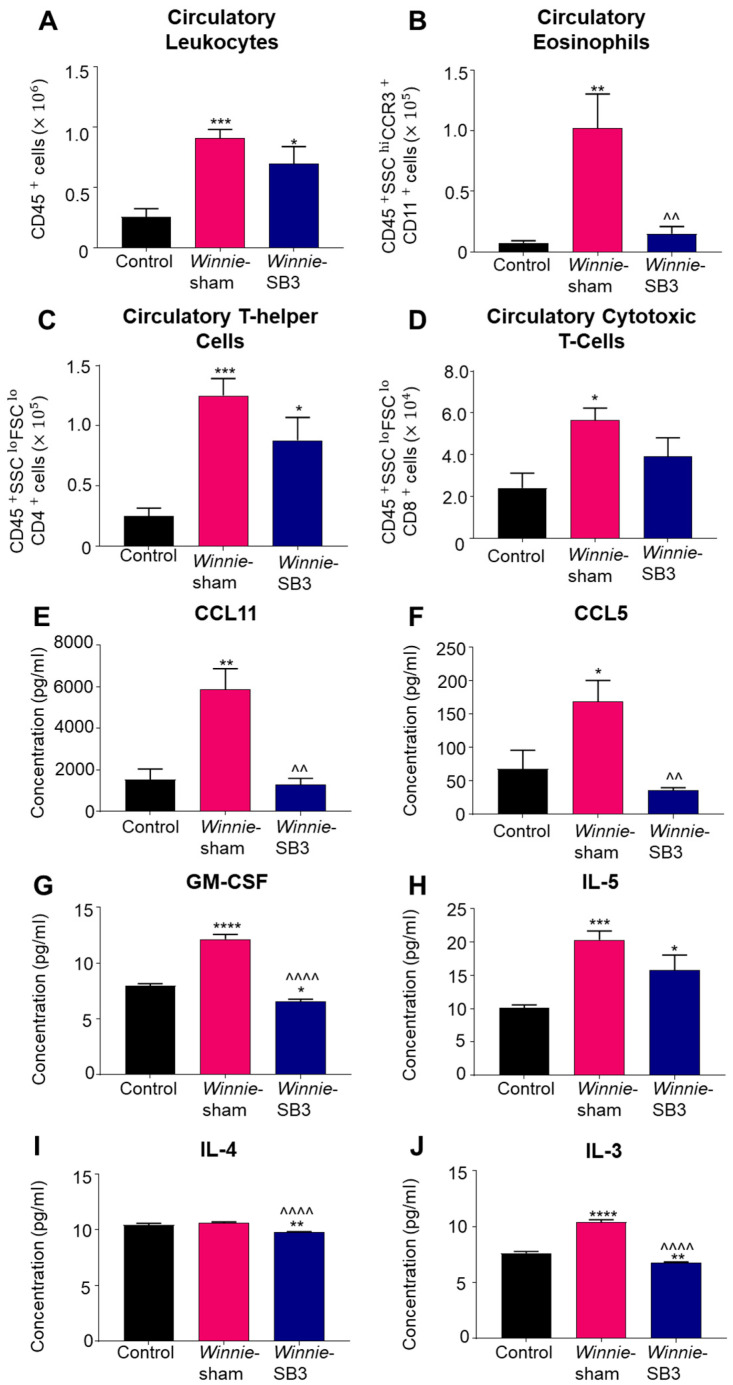
The effects of SB3 treatment on circulating leukocytes and serum levels of eosinophil-associated analytes. (**A**–**D**) The number of infiltrating CD45^+^ leukocytes, CD45^+^SSC^+^CCR3^+^CD11b^+^ eosinophils, CD45^+^SSC^LO^FSC^LO^CD4^+^ T helper, and CD45^+^SSC^LO^FSC^LO^CD8^+^ cytotoxic T cells measured in the blood sera from control, sham-treated, and SB3-treated *Winnie* mice (*n* = 6/group). (**E**,**F**) Concentrations of CCR3-associated chemokines, CCL11 and CCL5 (pg/mL), measured in the blood sera from control (*n* = 6), sham-treated *Winnie* (*n* = 6), and SB3-treated *Winnie* (*n* = 5) mice. (**G**–**J**) Concentration of eosinophil-associated cytokines GM-CSF, IL-5, IL-4, and IL-3 (pg/mL) quantified in the blood sera from control (*n* = 6), sham-treated *Winnie* (*n* = 6), and SB3-treated *Winnie* (*n* = 5) mice. Data expressed as mean ± SEM; * *p* < 0.05, ** *p* < 0.01, *** *p* < 0.001, **** *p* < 0.0001 compared to control mice; ^^ *p* < 0.01, ^^^^ *p* < 0.0001 compared to sham-treated *Winnie* mice.

Changes in the number of leukocytes, eosinophils, and T cell subsets were discerned in peripheral blood samples from control, sham-treated *Winnie,* and SB3-treated *Winnie* mice. An increase in circulating CD45^+^ leukocytes was noted in sham-treated *Winnie* mice compared to controls (*p* < 0.001, Table 2; Figure 5A). Treatment with SB3 did not reduce the number of CD45^+^ leukocytes in peripheral blood, which was significantly increased when compared to controls (*p* < 0.05). Eosinophil profiles were consistent with and without a marker for Siglec-F (Supplementary Figure S1). Studies have suggested that a combination of two or more markers for CCR3, CD11b, and/or Siglec-F can delineate eosinophil subsets [26]. Accordingly, isolated blood cells were labeled with antibodies against CD45^+^SSC^HI^CCR3^+^CD11b^+^, to discern changes in the blood eosinophils. Increased cell counts for CD45^+^SSC^HI^CCR3^+^CD11b^+^ eosinophils in the peripheral blood from sham-treated *Winnie* mice (*p* < 0.01 compared to controls) were significantly attenuated by SB3 treatment (*p* < 0.01 compared to sham-treated *Winnie* mice, Table 2; Figure 5B). There was an increase in the number of CD45^+^SSC^LO^FSC^LO^CD4^+^ T cells in sham- (*p* < 0.001) and SB3-treated (*p* < 0.05) *Winnie* mice in the peripheral blood compared to control mice (Table 2; Figure 5C). The number of circulatory CD45^+^SSC^LO^FSC^LO^CD8^+^ T cells was also increased in *Winnie* mice receiving sham treatments compared to controls (*p* < 0.05, Table 2; Figure 5D).

Concentrations of eosinophil-associated chemokines (CCL11 and CCL5) and cytokines IL-3, IL-4, IL-5, and GM-CSF, which play important roles in eosinophil maturation, chemotaxis, and activity, were measured in sera. Significant increases in concentrations of both CCL11 (*p* < 0.01, *n* = 6) and CCL5 (*p* < 0.05, *n* = 6) chemokines were evident in sham-treated *Winnie* mice compared to controls (*n* = 6; Table 3; Figure 5E,F). SB3 treatment attenuated concentrations of CCL11 and CCL5 to control levels (*p* < 0.01 for both). GM-CSF concentration was significantly increased in sham-treated *Winnie* mice (*p* < 0.0001, *n* = 6) compared to controls (*n* = 6; Table 3; Figure 5G). SB3 treatment reduced GM-CSF concentration (*p* < 0.0001, *n* = 5) compared to sham-treated *Winnie* mice and controls (*p* < 0.05). IL-5 was increased in both sham-treated (*p* < 0.001, *n* = 6) and SB3-treated (*p* < 0.05, *n* = 5) *Winnie* mice compared to controls (*n* = 6; Table 3; Figure 5H). SB3 treatment (*n* = 5) reduced IL-4 concentrations in both *Winnie* (*p* < 0.0001, *n =* 6) and control mice (*p* < 0.01, *n* = 6; Table 3; Figure 5I). A significant increase in IL-3 concentration in sham-treated *Winnie* mice (*p* < 0.0001, *n* = 6) compared to controls (*n* = 6; Table 3; Figure 5J) was attenuated following SB3 treatment (*n =* 5).

### 2.5. SB3 Treatments Did Not Affect Eosinophil Progenitor Cells in the Bone Marrow of Winnie Mice

**Figure 6:**
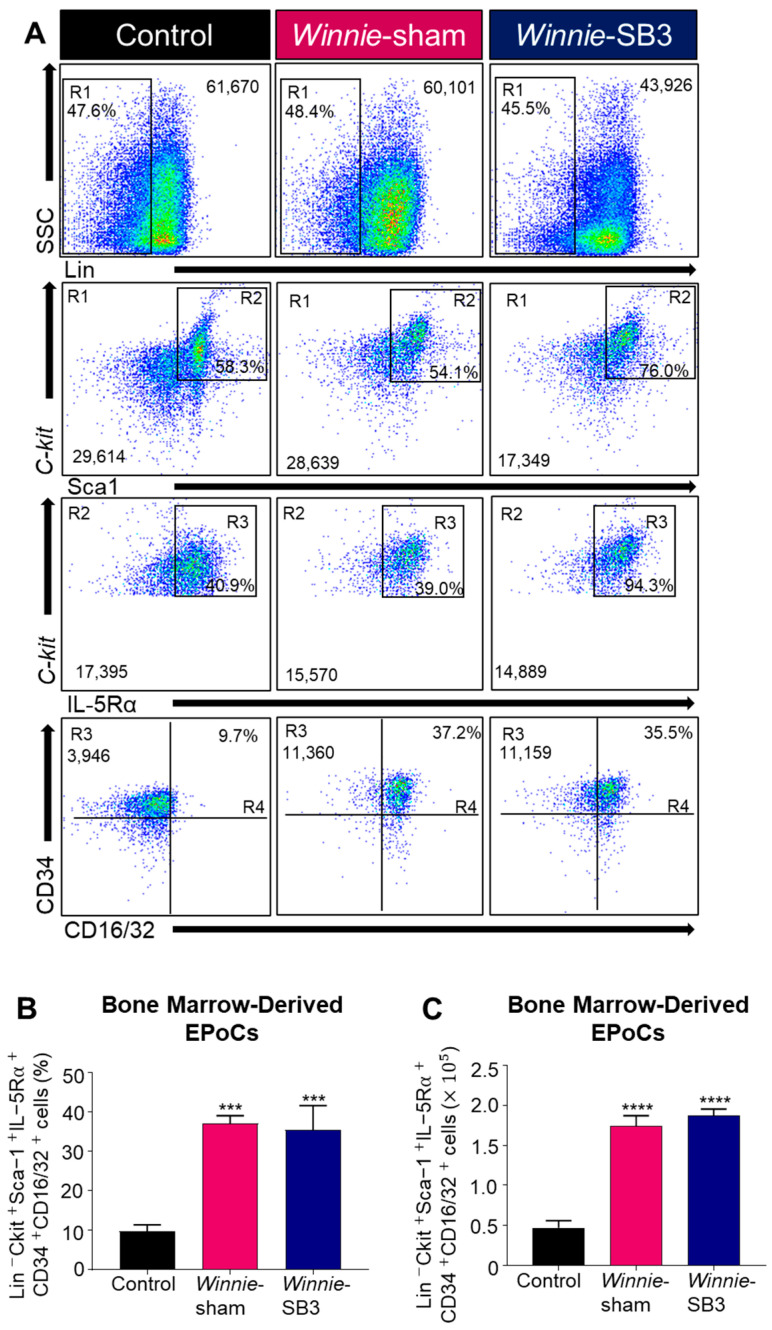
The effects of SB3 treatment on eosinophil progenitor cells (EPoCs) in bone marrow. (**A**) Gating strategy employed on viable cells: (R1) Lin^−^SSC^LO/HI^ was used to remove mature cell types, with subsequent gating on (R2) *C-kit*^+^Sca1^+^, (R3) *C-kit*^+^IL-5Rα^+^, and (R4) CD34^+^CD16/32^+^ to discriminate EPoCs in the bone marrow. Proportions (**B**) and cell counts (**C**) for the Lin^−^*C-kit*^+^Sca1^+^IL-5Rα^+^CD34^+^CD16/32^+^ EPoCs (*n* = 7/group). Data are expressed as mean ± SEM; *** *p* < 0.001, **** *p* < 0.0001 compared to control mice.

Eosinophil progenitor cells (EPoCs) were isolated from the bone marrow and characterized by Lin^−^*C-kit*^+^Sca1^+^IL-5Rα^+^CD34^+^CD16/32^+^ expression (Figure 6A). Increases in both proportions (*p* < 0.001) and cell counts (*p* < 0.0001) of EPoCs were noted in the bone marrow from sham-treated *Winnie* mice compared to controls (Table 2, Figure 6B,C) but were not altered following SB3 treatment.

## 3. Discussion

Eosinophils are multifunctional leukocytes that play a pivotal role in inflammatory cascades within the gastrointestinal tract [27]. In the present study, successful inhibition of eosinophil accumulation in the inflamed colon was achieved via targeting CCR3 receptors with a potent antagonist SB328437 (SB3), in the *Winnie* murine model of spontaneous chronic colitis. Effects of in vivo treatment on eosinophils and their progenitor cells were assessed in the colon, spleen, blood circulation, and bone marrow. Additionally, chemokines and cytokines that are involved in eosinophil infiltration and activities were evaluated in the sera from sham-treated, SB3-treated *Winnie,* and control mice.

This is the first study that provides refined insight into the anti-eosinophil and inflammatory effects of SB3 on the eosinophil-accumulating factors (chemokines and cytokines) involved in the migration of mature and naïve eosinophil populations to both local and systemic sites of inflammation. Animal models of IBD have been extensively utilized to unravel pathophysiological processes and to define molecular targets for IBD treatment [74,75]. In this study, we used the *Winnie* murine model of spontaneous chronic intestinal inflammation. *Winnie* mice show robust, progressive, and severe chronic colitis by 9–12 weeks of age, with inflammation-associated biomarkers similar to those noted in patients with UC [67,70]. Inability to gain body weight, chronic diarrhea, and rectal bleeding are prominent symptoms in the *Winnie* mouse model of colitis [66,67,73,76]. The progressive nature of intestinal inflammation and persistent symptoms apparent in *Winnie* mice are similar to clinical manifestations of human IBD [77]. Our results demonstrated that SB3 treatment alleviated colitis-associated symptoms and parameters of intestinal damage, such as morphological changes in the epithelium, presence of ulcerations, leukocyte infiltration, and irregular colonic crypts in the colons from *Winnie* mice. These results are consistent with previous findings in other models of intestinal and airway inflammation demonstrating that targeting the CCR3 ligand axis had anti-inflammatory effects and restored tissue integrity [13,59,62,78,79]. Additionally, our previous study in the TNBS-induced model of acute colitis in guinea-pigs demonstrated that treatment with a CCR3 receptor antagonist, SB328437, attenuated eosinophil infiltration to the enteric nervous system, prevented enteric neuropathy, and ameliorated colonic dysmotility [13]. No adverse effects of SB3 treatment have been observed in our studies.

Our results demonstrated that *Winnie* mice showed an increased number of infiltrating CD45^+^ leukocytes in the inflamed colon, which is consistent with previous reports [66,70,76]. Among the infiltrating leukocytes, persistent accumulation of eosinophils is considered a hallmark of intestinal inflammation during IBD pathogenesis [6,80]. Eosinophils have been regarded as more than “bystanders” as they contain granules that can produce, store, and release potent cytotoxic proteins, which have been previously detected in several biological fluids, gut lavage, and colonic biopsies acquired from patients with IBD [48,81,82,83,84]. During intestinal inflammation, activated eosinophils hamper pro-inflammatory cascades leading to host tissue toxicities [12,85,86]. In this study, we characterized an accumulation of CD45^+^SSC^HI^CCR3^+^CD11b^+^Siglec-F^+^ expressing eosinophils in the inflamed colon of *Winnie* mice. Our results demonstrated that SB3 treatment attenuated the accumulation of CD45^+^ leukocytes and, more specifically, CD45^+^SSC^HI^CCR3^+^CD11b^+^Siglec-F^+^ eosinophils in the inflamed colon. These findings are consistent with previous reports using a CCR3-specific antibody in a mouse model of spontaneous ileitis, as well as the SB3 treatment in our previous study in a TNBS-induced guinea-pig model of acute colitis [13,62]. In addition, our findings demonstrate that the infiltration of CD45^+^CCR3^+^ cells to the inflamed colon in *Winnie* mice was suppressed with SB3 treatment. The recruitment and activity of CCR3^+^ expressing cells, such as eosinophils and T cells, results in dissociation of the G-protein-coupled CCR3 receptor into G-α and G-βγ subunits, leading to activation of the Rho-guanosine-5’-triphosphate (GTP)ase pathway [87,88]. Selective inhibition of CCR3 receptors with SB3 has been shown to inhibit Rho activity, calcium (Ca^2+^) immobilization, and actin polymerization, which is pivotal for the recruitment and activation of eosinophils and T cells [89]. CCR3-induced eosinophil chemotaxis activates Rac2, which is a member of the Rho family of GTP-metabolizing proteins involved in regulating the actin cytoskeleton [90]. Furthermore, SB3 partially blocks CCL11 chemokine activity [89]. In vitro treatment of isolated eosinophils with SB3 suppressed CCR3 receptors, which weakened receptor-ligand binding affinity to CCL11 and CCL13-induced migration [89,90]. Additionally, activation of CCR3 receptors promotes eosinophil degranulation [33]. Thus, CCR3 receptors can be regarded as a viable target for counteracting the recruitment and activities of CCR3^+^ expressing cells within the inflamed intestine.

In the present study, an increase in CD4^+^ T helper cells was noted in the inflamed colons from *Winnie* mice. CD4^+^ T cells became polarized to Th1/Th2/Th17 subsets based on specific activation programs during intestinal inflammation [91]. Our results demonstrate that *Winnie* mice have an increase in the number of CD8^+^ T cells in the inflamed colon, which is also associated with chronic inflammation in human IBD and 2,4-dinitrobenzene sulfonic acid (DNBS)-induced mouse model of colitis [92,93,94]. During intestinal inflammation, the priming of CD8^+^ T cells, due to exposure to inflammatory mediators and antigens, results in relapses of colitis [92,95,96]. Activated CD8^+^ T cells are known to upregulate the expression of chemokine receptors, such as CCR3, CCR4, and CCR5, under specific conditions [97]. CCR3 receptors are responsible for the transmigration of eosinophils and polarization of Th2 T cells, as shown in inflamed colon samples from IBD patients [29]. In the human IBD and DSS-induced murine model of colitis, Th2 polarization regulates cytokine and chemokine profiles favorable for eosinophil recruitment during intestinal inflammation [29]. The results from this study demonstrated that SB3 treatment reduced the number of CD4^+^ T helper cells and CD8^+^ T cells in the inflamed colon to levels comparable to control mice. Therefore, it is plausible that SB3 treatment suppresses mechanisms involved in CD4^+^ and CD8^+^ T cell chemotaxis and cytolytic activities, which are yet to be explored during colitis.

Our results demonstrated a reduction in the spleen size in *Winnie* mice, which was not attenuated by SB3 treatment. Changes in secondary lymphoid organs can alter their roles in the filtration and storage of lymphocytes [70]. The organized compartments of the spleen include the red and white pulp regions, separated by the marginal zone [98]. In IBD pathogenesis, variations in the size of the spleen result in complicated inflammatory processes, leading to high rates of colitis [99,100]. In our study, the reduction in the spleen size in both sham-treated and SB3-treated mice was not accompanied by alterations in the total number of leukocytes compared to the control. The major cells of the spleen include T and B lymphocytes, macrophages, and dendritic cells but only a small fraction of eosinophils are known to reside within the spleen [98,101]. CD4^+^ T cells were increased in spleens from *Winnie* mice. This cell type is largely restricted to the white pulp regions that include the T and B cell zones, known as follicles [98]. Cell populations in the red pulp are mainly comprised of macrophages responsible for filtering the blood and recycling iron from old red blood cells [98]. In the IBD model, transplantation of small pieces of the gut from immunocompetent syngeneic donors to severe combined immunodeficient (SCID) mice results in the accumulation of CD4^+^ T cells within the spleen [102]. It was reported that CD4^+^ T cells exposed to the major antigen and inflammatory mediators in the inflamed colon were re-circulated back into the spleen [102], which might be a potential mechanism for splenic CD4^+^ T cell accumulation in *Winnie* mice. The treatments with SB3 might have reduced the trafficking of CCR3-mediated CD4^+^ T cells to the spleen. No differences in the eosinophil population were observed in the spleens between all experimental groups. Similar to our results, mice infected with *N.* *brasiliensis* demonstrated no changes in BrdU^+^Siglec-F^+^ eosinophil turnover in the spleen compared to untreated mice, suggesting that primary migration occurs at the site of inflammation or infection [103]. On the contrary, an increase of CCR3^+^SSC^HI^ eosinophils in the spleen was observed in IL-5 transgenic mice subjected to DSS-induced colitis [104]. Moreover, SB3 treatment did not affect the size and overall cellular populations of the spleen, postulating that this may hinder the efficiency of the treatment for other cell types; this warrants further attention.

Leukocytes migrate to sites of inflammation, infection, and damage through chemoattractant gradients during IBD [1]. In the present study, an increase in the total number of CD45^+^ leukocytes, CD45^+^SSC^HI^CCR3^+^CD11b^+^Siglec-F^+^ eosinophils, and CD4^+^ and CD8^+^ T lymphocytes were observed in the blood samples from sham-treated *Winnie* mice. However, SB3 treatment only reduced peripheral CD45^+^SSC^HI^CCR3^+^CD11b^+^Siglec-F^+^ eosinophils, confirming that SB3 is a highly specific antagonist of the CCR3 receptors involved in the transmigration of eosinophils.

Eosinophils account for only 5% of the total cells within the circulation [105]; however, IL-3, IL-5, and GM-CSF cytokines prime eosinophils in the peripheral blood [18,19,106,107]. We demonstrated an increase in the concentration of eosinophil-associated chemokines, CCL11 and CCL5, and cytokines, GM-CSF, IL-5, and IL-3, identified in the sera of *Winnie* mice. These findings are similar to reports in IBD patients that demonstrated increased blood sera levels for eosinophil-associated cytokines, GM-CSF and IL-5, and a chemokine RANTES (CCL5) compared to healthy controls [108]. However, an increase in blood sera concentration for eotaxin and IL-4 was detected exclusively in UC patients, suggesting their role in the differential diagnosis from patients with CD [108]. Th2-released cytokines, such as IL-5 and IL-13, are prominent in UC patients [109]. Th2 cell polarization during intestinal inflammation is allied with eosinophil recruitment, activation, and maturation [29]. CCR3 receptor expression and activity on Th2 cells facilitate the release of cytokines involved in priming for eosinophil activations. Cytokines (IL-5, IL-4, IL-3, GM-CSF, and IL-13) [106,107] and chemokines (CCL11 and CCL5) [34,35] released by CD4+ Th2 cells have profound effects on eosinophil activation and transmigration [110]. CD4^+^ Th2 cells are major contributors to the production of IL-4, which promotes eosinophil survivability and chemotaxis via increasing expression of eotaxin chemokines [111]. IL-4 and IL-5 released by differentiated Th2 cells facilitated the activation of eosinophils in in vitro transwell assays [112]. High levels of IL-5 prompting activation of eosinophils in the inflamed colon were measured in biopsies from IBD patients [11,113,114]. Moreover, IL-5 blockade hindered eosinophil activities and ameliorated intestinal inflammation in a model of chronic colitis induced by T cell transfer into *Rag*^−/−^ mice [115]. Although IL-5 has the capacity to modulate eosinophil activities, chemokines are relatively selective in facilitating eosinophil trafficking [6,116]. These data provide evidence that CCR3 receptors are involved in Th2 polarization and the release of chemokines and cytokines that are the main contributors to the regulation of eosinophil recruitment [78,89,112]. Our results demonstrate that the treatment of *Winnie* mice with SB3 attenuated eosinophil-associated regulatory proteins CCL11, CCL5, GM-CSF, IL-4, and IL-3 in the serum, but had no effect on IL-5. This may imply that the production of IL-5 is not limited to hematopoietic-derived cells such as eosinophils and CD4^+^ Th2 cells; IL-5 may also be produced by non-hematopoietic lineages, as has been suggested in previous studies [117,118]. Such cells include mast cells that have been reported to be abundant in the sites of active mucosal inflammation further contributing to the expanding damages to the intestines seen in UC and CD patients [119]. Intestinal mast cells have been considered active contributors to the production of the pleiotropic cytokine IL-5 during the pathogenesis of IBD [119]. Additionally, intestinal mast cells isolated and purified from patients with IBD express IL-5, which was not found in healthy controls [120]. These findings suggest that apart from T lymphocytes and eosinophils, activated intestinal mast cells are involved in IL-5 production during intestinal inflammation [120]. Moreover, the non-cytotoxic innate lymphoid cells (ILCs) are classified into three groups: ILC1s, ILC2s, and ILC3s, mirroring CD4^+^ T helper cell functions [121]. Activated mast cells can directly stimulate ILC2s to drive an IL-5-associated Th2 response [121]. ILC2s are highly dependent on the transcription factors GATA-binding protein 3 (GATA3) and retinoic acid receptor-related orphan receptor-alpha (RORα), which facilitate the secretion of IL-4, IL-5, IL-9, and IL-15 [121]. However, no differences in the amount of ILC2s were found in the intestine and blood samples from healthy controls and patients with IBD, suggesting that they may have limited roles during intestinal inflammation [122]. Further studies are required to map out the cytokine and chemokine release by all associated leukocytes and non-leukocytes in the *Winnie* model of IBD, with and without SB3 treatment.

Eosinophil recruitment requires specific stimuli to evoke eosinopoiesis in bone marrow [15,16,17]. In this study, we analyzed eosinopoiesis in the bone marrow of sham-treated and SB3-treated *Winnie* mice. Eosinophil progenitor cells (EPoCs) are typically isolated based on their expression for IL-5Rα^+^, *c-kit*^+^, and CD34^+^,as they are derived from hematopoietic stem cell (HPSC) precursors [123,124,125]. It has been described that the transcription factor GATA-1 is crucial for granulocyte/macrophage progenitor cells to transpose into EPoCs [123]. However, expansion of the pool of EPoCs is not only subject to GM-CSF but also heavily influenced by IL-5 and IL-3. These cytokines involved in eosinopoiesis are regarded as eosinophil hematopoietic cytokines (EPHCs) [19]. EPHCs are vital for EPoC commitment and maturation, which generates the appropriate receptor expression on eosinophils that prompts them to undergo transmigration during inflammatory pathologies [20,21]. Our data demonstrated an increase in Lin^−^*C-kit*^+^Sca1^+^IL-5Rα^+^CD34^+^CD16/32^+^ EPoCs in the bone marrow, which coincided with elevated serum levels of the EPHCs, IL-5, IL-3, and GM-CSF in sham-treated *Winnie* mice. GM-CSF is a potent activator of eosinophils involved in increasing eosinopoiesis in bone marrow [115]. In vivo treatments with a monoclonal antibody against GM-CSF in a model of chronic colitis induced by T cell transfer into *Rag*^−/−^ mice, reduced EPoCs in bone marrow and dampened eosinophil chemotaxis to the inflamed colon [115]. In vivo and in vitro studies have demonstrated that Th2-mediated cytokines induce the migration of CD34^+^ progenitor cells [104,126,127]. Furthermore, these CD34^+^ progenitor cells had an increase in mRNA expression for CCR3 receptors, potentially leading to the differentiation of eosinophil lineage [127]. As EPoCs differentiate into mature eosinophil lineages, CD34^+^ expression diminishes, due to activation by factors inducing eosinopoiesis, inclusive of IL-3, IL-5, and GM-CSF [128]. In our study, the lack of effects of SB3 treatment on the number of EPoCs in the bone marrow of mice with chronic colitis coincided with elevated IL-5 levels in their serum after treatment. IL-5 has been described as priming CD34^+^ progenitor cells, which induce changes to the transcriptional profile suitable for eosinophil lineage progression [129]. Therefore, the considerable interest in targeting eosinophil populations resulted in the implementation of the following treatments: (1) anti-common beta (β) chain therapy, which targets IL-5, IL-3, and GM-CSF-associated growth factors; (2) anti-chemotaxis therapy, which inhibits the activity of migration via targeting chemokines and their receptors; and (3) anti-cytokine specific therapy, which uses monoclonal antibodies against IL-4, IL-5, and IL-13, which influence eosinophil differentiation and activation in eosinophil-associated diseases such as asthma [128]. Conflicting reports have demonstrated that targeting one of the above eosinophil-associated targets had a profound effect on EPoC populations [128]. However, targeting the CCR3 receptors demonstrated no changes to EPoCs [130]. A randomized, double-blind study investigating the efficacy of a potent small-molecule CCR3 receptor antagonist GW766994 on infiltrating mature and immature eosinophils demonstrated that inhibition of CCR3 receptor activity with GW66994 had no effect on EPoCs isolated from peripheral blood and sputum in asthma patients [130]. These results are consistent with our findings and provide evidence that targeting CCR3 receptors does not alter the elevated level of eosinophil precursors. Alternatively, studies that have used monoclonal antibodies against common β chains and CCR3 receptors have demonstrated a dampened number of bone marrow-derived EPoCs in eosinophil-associated conditions such as asthma and ileitis, respectively [65,131]. Therefore, the ineffectiveness of SB3 treatment on the high level of IL-5 might be a possible explanation for the elevated level of EPoCs in our model; however, further studies on the mechanism behind IL-5 priming on EPoCs in bone marrow are warranted.

## 4. Methods and Materials

### 4.1. Animals

*Winnie* mice (12 w.o; 16–30g; males and females, *n* = 32) were obtained from the Victoria University Werribee Animal Facility (Melbourne, VIC, Australia). C57BL/6 (12 w.o; 20–30 g; males and females, *n* = 16) used as healthy controls were obtained from the Animal Resource Centre (Perth, Australia). All animals were housed at Victoria University’s Western Centre for Health, Research and Education campus (Melbourne, VIC, Australia) on a 12-h day/night cycle, in a temperature-controlled facility. All mice had free access to water and food, with all efforts made to reduce any animal suffering. All experimental procedures adhered to the guidelines of the Australian National Health and Medical Research Council (NHMRC) and were approved by the Victoria University Animal Experimentation Ethics Committee (AEETH: 17/016).

### 4.2. Administration of SB328437 (SB3) and Anesthesia

A potent CCR3 antagonist, SB328437 (SB3; SML0207; Sigma-Aldrich, St. Louis, MO, USA) was administered intraperitoneally (i.p.) in *Winnie* mice at a dose of 13.75 mg/kg dissolved in the vehicle solution: cremophor (2%)/ethanol (2%)/sterile water (96%), which had previously been demonstrated to attenuate eosinophil recruitments and provide neuroprotective properties in a guinea-pig model of TNBS-induced colitis [13,132]. Mice received 3 injections per week for 2 weeks, to determine the efficacy of SB3. *Winnie*-sham treated animals received a vehicle excluding SB3. All injections were performed using a 30G needle; the injected volume was calculated per body weight, with a maximum of 200 µL per injection. On day 14, animals were administered an overdose of pentobarbital (1:16 dilution, 100 µL/20g, 30G needle, i.p.) to anesthetize them prior to performing cardiac puncture for blood collection. Blood was collected for flow cytometry and eosinophil-associated analytes in the serum. Subsequently, the entire colon, spleen, and bone marrow tissues were harvested for flow cytometry. Distal colon samples were collected for histological assessment and immunohistochemical analysis.

### 4.3. Disease Activity

Animals were monitored over the 14-day course of treatment for clinical symptoms. Body weights were recorded daily, with any changes to body weight calculated as a percentage of change from day 1 of the treatment. Fresh fecal samples were collected one day prior to culling to measure fecal water content. Immediately after culling, the whole colon and spleen were excised, changes in their weights were measured, and histopathological analysis of the colon sections was performed. The weight of whole colon samples included fecal content, to capture inconsistency in both the colon mass and fecal water retention indicative of diarrhea.

### 4.4. Blood, Colon, Spleen, and Bone Marrow Single Cell Suspensions

Approximately 1 mL of blood was collected with a 26G needle into BD vacutainer plastic K2EDTA tubes (BD Diagnostic, 367839, Franklin Lake, NJ, USA). Blood samples were immediately diluted in 1 mL of 1× phosphate-buffered saline (PBS) and transferred delicately to a FACS tube containing 800 µL of Ficoll-Paque™ PLUS (GE Healthcare, 17-1440-03, Chicago, IL, USA). Blood samples were centrifuged at 400× *g* at 23 °C, with an acceleration of 7, and deceleration of 0, for 40 min, to isolate buffy coats, high dense granulocytes, and plasma. Buffy coats, plasma, and high dense cells were collected in 1 mL of FACS buffer (1× PBS, 5% FCS, 0.01% sodium azide). The whole colon was dissected, from the caecum to the anus, and pinned into a silicon-lined petri dish (Slygard^®^ 184, Dow Corning, Midland, MI, USA) containing α-MEM media. The colon was cleaned thoroughly, removing surrounding adipose tissue and fecal matter from the lumen. Briefly, the colons were washed in α-MEM media 3 times prior to mechanical and chemical dissociation. Under a light microscope, microdissection of tissues was performed; tissues were cut into fragments before being incubated with a digestive enzyme, Collagenase Type 4 (Thermofisher, 17104019, Waltham, MA, USA) (0.1% *w*/*v* in 2 mL of α-MEM) for a total of 2 h at 37 °C. Subsequent mechanical dissociation was performed to colon tissue fragments in 30-min intervals via aspirating samples with a sterile pipette tip. Once digestion was complete, 1 mL of FACS buffer was added to the cell suspension prior to centrifugation at 1500 RPM, 4 °C for 5 min, and pellets were resuspended in 1 mL of FACS buffer to create a cell suspension. Spleens were harvested, cleared of any excess adipose tissue, and transferred into a glass Petri dish containing 2 mL of FACS buffer to perform mechanical dissociation of the tissue. Bones from both the left and right hind limbs were collected using an incision from the midline of the abdomen to above the hip and, subsequently, to the ankle to detach the skin and muscle layers. The femurs were dislodged from the hip joint and cut at the lesser trochanters and adductor tubercles. Bone marrow was flushed from the femur with a 23-G needle containing FACS buffer into a 50-mL Falcon® tube. All cells were subsequently filtered through a 70-µm Falcon® cell strainer into a 50-mL Falcon® tube and centrifuged at 1500 RPM, 4 °C, for 5 min. Cells were briefly incubated with 1 mL of 1× red blood cell lysis buffer (BD Pharm Lyse™, Franklin Lake, NJ, USA) for 10 min at room temperature in the dark. Once the incubation was completed, 1 mL of FACS buffer was added to cells prior to centrifugation at 1500 RPM, 4 °C for 5 min, and pellets were resuspended in 1 mL of FACS buffer to create a cell suspension. Cell counts were generated using a haemocytometer cell count. Cells were diluted in FACS buffer (1:10), in which 10 µL of the cells were treated with 10 µL of Trypan Blue (1:1) to quantify 1 × 10^6^ cells per sample.

### 4.5. Flow Cytometry Analysis

Two panels were employed in this study to characterize changes in leukocyte populations with a specific focus on eosinophils. Panel 1 antibodies included cell surface markers FITC anti-mouse CD45 (1:400, BD Pharmingen^TM^, 553079, Franklin Lake, NJ, USA), PE-Cy7 anti-mouse CD11b (1:400, BD Pharmingen^TM^, 552850, Franklin Lake, NJ, USA), AF647 anti-mouse CCR3 (1:50, BD Pharmingen^TM^, 557974, Franklin Lake, NJ, USA), BV421 anti-mouse Siglec F (1:100, BD Horizon^TM^, 562681, Franklin Lake, NJ, USA), BV480 anti-mouse CD4 (1:400, Horizon^TM^, 565634, Franklin Lake, NJ, USA) and APC-Cy7 anti-mouse CD8a (1:200, Pharmingen^TM^, 557654, Franklin Lake, NJ, USA) to discern leukocytes, eosinophils, T-helper, and cytotoxic T cells. To isolate eosinophil progenitor cells in bone marrow cells, panel 2 contained antibodies against V500 anti-mouse Ly-6A (SCA-1) (1:200, BD Horizon^TM^, 561228, Franklin Lake, NJ, USA), APC Ms lineage cocktail (contains mouse antibodies against CD3e; CD11b; CD45R/B220; Ly-76 and Ly-6G and Ly-6C; 20 µL/volume, Pharmingen^TM^, 558074, Franklin Lake, NJ, USA), BB515 anti-mouse CD117 (*c-kit*) (1:100, BD Horizon^TM^, 564481, Franklin Lake, NJ, USA), PE anti-mouse CD125 (IL-15Rα) (1:50, BD Pharmingen^TM^, 558488, Franklin Lake, NJ, USA), BV421 anti-mouse CD34 (1:200, BD Horizon^TM^, 562608, Franklin Lake, NJ, USA), and PE-Cy7 anti-mouse CD16/CD32 (1:50, BD Pharmingen^TM^, 560829, Franklin Lake, NJ, USA).

Cells were aliquoted into 5-mL FACS tubes containing 1.0 × 10^6^ cells per sample and incubated with Fc blocker (1:100, BD Pharmingen^TM^, 553142, Franklin Lake, NJ, USA) for 20 min at 4 °C [133]. Subsequently, cells were incubated with 200 µL of the appropriate panel including for 45 min at 4 °C in the dark. The cells collected from the colon, spleen, and blood were incubated with panel 1. Cells obtained from the bone marrow were incubated with panel 2. Cells were washed with FACS buffer twice before being resuspended in 400 µL of FACS buffer and 20 µL of 7AAD viability solution (BD Via-Probe^TM^, 555815, Franklin Lake, NJ, USA), to determine cellular viability prior to the analysis. Prior to analysis, all cells were gated to discriminate doublets and 7AAD^-^ cells, to remove dead and unviable cells (Supplementary Figure S2).

The labelled cells were identified and quantified with the use of flow cytometry (BDFACSAria III, BD Bioscience, Franklin Lake, NJ, USA), with data being acquired using FACS DIVA v6.1 (BD Bioscience, Franklin Lake, NJ, USA) software. For each sample, 100,000 events were collected. Data were exported to Flow Cytometry Standard (FCS) 3.0 files and subsequently analyzed using FlowJo v10.1r5 (Treestar, CA, USA). As each antibody marker produces distinctive emission spectra, they can be individually identified, with every single marker being representative of a specific cell population within the colon, blood, spleen, and bone marrow niches. For all experiments, single color compensation controls using (BD^TM^ Anti-rat and Anti-hamster Ig_K_/negative control compensation particle set) and fluorescence minus one (FMO) were employed to optimize PMT voltages and calculate spectral overlap (if any).

### 4.6. Tissue Collection and Preparation

Distal colon tissues were harvested and placed in oxygenated physiological saline in a silicon-lined petri dish. Colons were opened by cutting along the mesenteric border, pinned mucosal side facing up, and submerged in Zamboni’s fixative (2% paraformaldehyde, 0.2% picric acid) overnight at 4 °C. Briefly, fixed colon samples were washed with dimethyl sulfoxide (DMSO; Sigma-Aldrich, Castle Hill, NSW, Australia) (3 × 10 min) and phosphate-buffered saline (PBS) (3 × 10 min). Full-thickness distal colon samples were pinned without stretching and fixed as above, placed into 50:50 optimum cutting temperature (OCT) compound (Tissue Tek, Torrance City, CA, USA), and were frozen in liquid nitrogen-cooled isopentane and OCT. Tissues were stored at −80 °C until cryo-sectioned (20 µm) onto glass slides for immunohistochemistry (IHC), to investigate changes to the CCR3-immunoreactive (IR) and CD45-IR leukocytes.

### 4.7. Histology and Immunohistochemistry

Paraffin-embedded samples were sectioned at 10 µm, cleared, and rehydrated in graded ethanol concentrations for standard hematoxylin and eosin (H&E) staining, as previously described [134]. A histological grading system determined structural damage to the colon from the following parameters: deviant crypt architecture (0–3), ulcerations (0–3), leukocyte infiltration (0–3), and epithelial damage (0–3) [67]. An average of 3 randomly selected areas of 500 µm^2^ per section were analyzed.

Immunohistochemical studies were performed as described previously [13,72]. Preparations were exposed to 10% normal donkey serum (NDS) (Merck Millipore, North Ryde, NSW, Australia) at room temperature for 1 h prior to washing with 1× PBS + Triton X (0.1%, 1× PBS-T) (3× 10 min). Sections were incubated with a primary antibody, rabbit anti-CD45 (1:1000; Ab10558, Abcam, Melbourne, VIC, Australia), overnight. Sections were washed with 1× PBS-T (3 × 10 min) and then incubated for 2 h with fluorophore-conjugated antibodies, donkey anti-rabbit Alexa Fluor 488 (1:500, Jackson Immunoresearch, West Grove, PA, USA) and AF647 rat anti-mouse CCR3 (1:50, BD Pharmingen^TM^, 557974, Franklin Lake, NJ, USA). All preparations were stained with 4′,6-diamidino-2-phenylindoledihydrochloride (DAPI) to label cell nuclei, prior to mounting on glass slides with the fluorescence mounting medium (DAKO, North Sydney, NSW, Australia).

### 4.8. Imaging and Quantitative Analysis

Histopathological changes were discerned in H&E-stained colon cross-sections with a Zeiss AxioImager microscope. H&E-stained sections were captured using the MetaSystems Metafer program and VSlide software stitched images together. All H&E-stained slides were coded, with the analysis performed blind. Immunolabeled specimens were visualized using a Nikon Eclipse Ti multichannel confocal laser scanning system (Nikon, Minato-ku, Tokyo, Japan). A combination of specific fluorophores including DAPI (excitation wavelength 405 nm), Alexa Fluor 594 (excitation wavelength 559 nm), and Alexa Fluor 488 (excitation wavelength 499 nm) was used. Z-series images were obtained at a nominal thickness of 0.5 µm (512 × 512). All analyses were performed blind using ImageJ software (National Institute of Health, Bethesda, MD, USA). Colon cross-sections were co-labelled with DAPI nuclear stain, to quantify the number of CCR3-IR and CD45-IR cells infiltrating the distal colon from 4–5 randomly captured images per specimen at ×40 magnification. All images were captured at an equal acquisition, exposure-time conditions, calibrated to a standard minimum baseline fluorescence, and converted to binary for analysis of density measured as a percentage (%) on ImageJ software.

### 4.9. Cytokine Analysis

Blood samples were collected with a 26-G needle and transferred into Eppendorf tubes without anticoagulants. The blood samples were centrifuged at 15 G for 15 min at room temperature. Serum samples were collected, aliquoted, and stored at −80 °C until use. Concentrations of 6 eosinophil-associated chemokines and cytokines were measured in serum samples by commercially available capture and detection antibodies in a Bio-Plex Pro Reagent Kit using a 6-plex kit (Bio-Rad, 12002798, Melbourne, VIC, Australia), which included the following analytes: eotaxin (CCL11), RANTES (CCL5), granulocyte-macrophage colony-stimulating factor (GM-CSF), interleukin (IL)-3, IL-4, and IL-5. The appropriate analyte standard blanks and samples were aliquoted into a 96-well plate containing antibodies that were attached to fluorescent-labelled microbeads. The samples were incubated in the dark at room temperature on an orbital shaker at 300 RPM for 30 min. The plate was washed 3 times, a detection antibody was added to each well, and was incubated in the dark at room temperature on an orbital shaker at 300 RPM for 30 min. Following 3 washes with 1× washer buffer, streptavidin-phycoerythrin was added to each well and the plate was incubated in the dark at room temperature on an orbital shaker at 300 RPM for 10 min. The beads were resuspended in 125 µL of a buffer prior to detection and quantification of capture beads using the BioPlex200 Multiplex System platform. Data were automatically analyzed cytokine response minus the background (pg/mL) of each treatment from 3 replicate wells using the Bio-Plex Manager 6.1 software.

### 4.10. Statistical Analysis

Data were analyzed using one-way ANOVA for multiple group comparison, followed by a Tukey–Kramer post hoc test (GraphPad Prism v9.0). Data are presented as mean ± standard error of the mean (SEM), if not specified otherwise. Variable *n* denotes the number of animals used for each experimental group and differences were considered statistically significant at a *p* value < 0.05.

## 5. Conclusions

Our study is the first to characterize local and systemic eosinophils and determine changes to their regulatory molecules in the murine *Winnie* model of chronic colitis. This study demonstrated that targeting eosinophils via the CCR3 axis has anti-inflammatory effects in the inflamed intestine. Treatment with SB3 in *Winnie* mice demonstrated reduced eosinophil accumulation in the inflamed colon and the circulation. Additionally, increased levels of eosinophil-associated cytokines and chemokines in blood sera were attenuated with SB3 treatment in *Winnie* mice. Suppressing CCR3-ligand interactions may inhibit the functional properties of eosinophils during intestinal inflammation. Therefore, utilizing clinically relevant animal models of IBD lays the foundation for understanding the role of eosinophils and their accumulating factors as potential end-point targets in treatments for IBD.

## Figures and Tables

**Table 1:** Changes to body weights of C57BL/6 control, sham-treated, and SB3-treated *Winnie* mice.

Day	Control *n* = 11	Sham-Treated *Winnie* Mice *n* = 12	SB3-Treated *Winnie* Mice *n* = 11
0	100.0 ± 0.0	100.0 ± 0.0	100.0 ± 0.0
1	103.4 ± 1.5	98.7 ± 0.4	99.3 ± 0.8
2	102.2 ± 2.3	98.5 ± 1.1	100.6 ± 0.8
3	100.9 ± 2.1	97.3 ± 1.2	102.7 ± 1.2
4	100.1 ± 1.7	97.3 ± 1.5	99.8 ± 1.0
5	100.8 ± 1.8	96. 2 ± 1.7	99.9 ± 1.0
6	102.0 ± 1.7	95.7 ± 1.6 *	99.3 ± 1.2
7	102.4 ± 2.01	95.5 ± 1.7 **	100.3 ± 1.1
8	103.9 ± 1.7	95.3 ± 1.7 ***	100.9 ± 1.2 ^
9	105.2 ± 1.9	94.2 ± 1.9 ****	100.7 ± 1.2 ^^
10	104.7 ± 1.8	93.1 ± 2.3 ****	100.1 ± 1.2 ^^
11	105.0 ± 1.6	94.1 ± 2.0 ****	100.3 ± 1.3 ^
12	105.8 ± 1.6	94.6 ± 1.8 ****	100.4 ± 1.2 *^
13	106.1 ± 1.3	94.3 ± 2.1 ****	99.9 ± 1.2 *^
14	106.7 ± 1.4	92.9 ± 1.8 ****	100.6 ± 1.5 *^^

* p < 0.05, ** *p <* 0.01, *** *p <* 0.001, **** *p <* 0.0001 when compared to C57BL/6 control mice; ^ *p* < 0.05, ^^ *p* < 0.01 when compared to sham-treated *Winnie* mice.

**Table 2:** Flow cytometry analysis discerning changes in the proportion and cell counts for leukocytes in the colon, spleen, blood, and bone marrow.

**Colon Single Cell Suspensions**				
Cell Population	Group/ Parameter	Control *n* = 8	Sham-Treated *Winnie* Mice *n* = 8	SB3-Treated *Winnie* Mice *n* = 8
CD45^+^ Leukocytes	Proportion	4.8 ± 0.6%	50.7 ± 7.8% ****	22.1 ± 2.1%, *^^^
	Cell Counts	4.3 × 10^5^ ± 6.0 × 10^4^	4.0 × 10^6^ ± 2.6 × 10^5^ ****	2.5 × 10^6^ ± 3.1 × 10^5^ ****^^^
CD45^+^SSC^HI^CCR3^+^CD11b^+^Siglec-F^+^ Eosinophils	Proportion	6.0 ± 1.6%	29.1 ± 4.5% ****	6.8 ± 1.6% ^^^^
	Cell Counts	7.9 × 10^3^ ± 2.4 × 10^3^	3.1 × 10^4^ ± 3.5 × 10^3^ ****	7.0 × 10^3^ ± 1.7 × 10^3^ ^^^^
CD45^+^SSC^LO^FSC^LO^CD4^+^ T helper lymphocytes	Proportion	5.1 ± 1.3%	24.5 ± 1.4% ****	6.5 ± 1.5% ^^^^
	Cell Counts	8.3 × 10^3^ ± 4.6 × 10^3^	2.4 × 10^5^ ± 7.4 × 10^4^ **	3.2 × 10^4^ ± 9.6 × 10^4^ ^^
CD45^+^SSC^LO^FSC^LO^CD8^+^ cytotoxic T lymphocytes	Proportion	9.3 ± 1.8%,	3.9 ± 1.2% *	4.9 ± 0.6%
	Cell Counts	2.0 × 10^4^ ± 1.0 × 10^4^	1.7 × 10^5^ ± 6.5 × 10^4^ *	6.0 × 10^2^ ± 1.3 × 10^2^ ^
**Spleen Single Cell Suspensions**				
Cell Population	Group/ Parameter	Control *n* = 7	*Winnie*-sham treated *n* = 7	*Winnie*-SB3 treated *n* = 7
CD45^+^ Leukocytes	Proportion	85.0 ± 12.1%	80.8 ± 5.7%	55.4 ± 8.3% ^
	Cell Counts	1.3 × 10^7^ ± 9.0 × 10^5^	9.9 × 10^6^ ± 1.9 × 10^6^	9.9 × 10^6^ ± 1.2 × 10^6^
CD45^+^SSC^HI^CCR3^+^CD11b^+^Siglec-F^+^ Eosinophils	Proportion	48.8 ± 6.4%	41.9 ± 13.0%	56.4 ± 9.6%
	Cell Counts	1.4 × 10^5^ ± 2.7 × 10^4^	1.5 × 10^5^ ± 4.5 × 10^4^	1.5 × 10^5^ ± 4.6 × 10^4^
CD45^+^SSC^LO^FSC^LO^CD4^+^ T helper lymphocytes	Proportion	5.1 ± 1.3%	22.3 ± 2.54% ****	6.5 ± 1.5% ^^^^
	Cell Counts	2.8 × 10^4^ ± 2.0 × 10^4^	5.9 × 10^5^ ± 1.5 × 10^5^ ***	5.1 × 10^4^ ± 1.4 × 10^3^ ^^^
CD45^+^SSC^LO^FSC^LO^CD8^+^ cytotoxic T lymphocytes	Proportion	9.3 ± 1.8%	3.9 ± 1.2% *	4.9 ± 0.6%
	Cell Counts	6.2 × 10^4^ ± 4.2 × 10^4^	7.0 × 10^4^ ± 2.1 × 10^4^	4.5 × 10^4^ ± 1.3 × 10^4^
**Isolated Blood Cell Suspensions**				
Cell Population	Group/ Parameter	Control *n* = 6	*Winnie*-sham treated *n* = 6	*Winnie*-SB3 treated *n* = 6
CD45^+^ Leukocytes	Proportion	25.4 ± 7.0%	90.7 ± 7.1% ***	69.4 ± 14.2% *
	Cell Counts	2.5 × 10^5^ ± 7.0 × 10^4^	9.1 × 10^6^ ± 7.1 × 10^4^ ***	6.9 × 10^5^ ± 1.4 × 10^5^ *
CD45^+^SSC^HI^CCR3^+^CD11b^+^ Eosinophils	Proportion	4.0 ± 1.0%	11.3 ± 2.7% *	2.0 ± 0.5% ^^
	Cell Counts	7.3 × 10^3^ ± 1.8 × 10^3^	1.0 × 10^5^ ± 2.8 × 10^4^ **	1.5 × 10^4^ ± 6.0 × 10^3^ ^^
CD45^+^SSC^LO^FSC^LO^CD4^+^ T helper lymphocytes	Proportion	12.3 ± 2.5%	14.1 ± 1.7%	22.3 ± 3.2% *
	Cell Counts	2.5 × 10^4^ ± 6.6 × 10^3^	1.3 × 10^5^ ± 1.4 × 10^4^ ***	1.4 × 10^5^ ± 3.2 × 10^4^ *
CD45^+^SSC^LO^FSC^LO^CD8^+^ cytotoxic T lymphocytes	Proportion	9.0 ± 1.0%	6.3 ± 0.6%	5.6 ± 0.4% *
	Cell Counts	2.4 × 10^4^ ± 7.1 × 10^3^	5.6 × 10^4^ ± 5.7 × 10^3^ *	3.9 × 10^4^ ± 9.0 × 10^3^
**Isolated Bone Marrow Cell Suspensions**				
Cell Population	Group/ Parameter	Control *n* = 6	*Winnie*-sham treated *n* = 6	*Winnie*-SB3 treated *n* = 6
Lin^−^*C-kit*^+^Sca1^+^IL-5Rα^+^CD34^+^CD16/32^+^ Eosinophil Progenitor Cells	Proportion	9.8 ± 1.7%	37.2 ± 1.7% ***	35.5 ± 6.2% ***
	Cell Counts	4.7 × 10^4^ ± 9.0 × 10^3^	1.7 × 10^5^ ± 1.3 × 10^4^ ****	1.8 × 10^5^ ± 8.4 × 10^3^ ****

* *p* < 0.05, ** *p* < 0.01, *** *p* < 0.001, **** *p* < 0.0001 when compared to control mice; *^ p* < 0.05, ^^ *p* < 0.01, ^^^ *p* < 0.001, ^^^^ *p* < 0.0001 when compared to sham-treated *Winnie* mice.

**Table 3:** Eosinophil-associated chemokines and cytokines in blood serum.

Analyte	Control *n* = 6	Sham-Treated *Winnie* Mice *n* = 6	SB3-Treated *Winnie* Mice *n* = 5
CCL11	1525.5 ± 517.5 pg/mL	5878.2 ± 993.4 pg/mL **	1294.7 ± 295.8 pg/mL ^^
CCL5	67.9 ± 28.1 pg/mL	169.1 ± 31.6 pg/mL *	35.9 ± 3.9 pg/mL ^^
GM-CSF	7.9 ± 0.2 pg/mL	12.1 ± 0.4 pg/mL ****	6.6 ± 0.2 pg/mL * ^^^^
IL-5	10.1 ± 0.5 pg/mL	20.3 ± 1.3 pg/mL ***	15.8 ± 2.3 pg/mL *
IL-4	10.4 ± 0.1 pg/mL	10.6 ± 0.1 pg/mL	9.8 ± 0.0 pg/mL ** ^^^
IL-3	7.6 ± 0.2 pg/mL	10.4 ± 0.2 pg/mL ****	6.7 ± 0.1 pg/mL ** ^^^^

* *p* < 0.05, ** *p* < 0.01, *** *p* < 0.001, **** *p* < 0.0001 when compared to control mice; ^^ *p* < 0.01, ^^^ *p* < 0.001, ^^^^ *p* < 0.0001 when compared to sham-treated *Winnie* mice.

## Data Availability

All data available upon request.

## Supplementary Materials

The following supporting information can be downloaded at: https://www.mdpi.com/article/10.3390/ijms23147780/s1.
