# Supplementary material for: Potent CCR3 Receptor Antagonist, SB328437, Suppresses Colonic Eosinophil Chemotaxis and Inflammation in the Winnie Murine Model of Spontaneous Chronic Colitis
Source: Int J Mol Sci. 2022 Jul 14;23(14):7780. doi: 10.3390/ijms23147780 (PMC9317166; doi:10.3390/ijms23147780)
Supplement: Supplementary file 1 [file ijms-23-07780-s001.zip › ijms-1745495-supplementary.pdf]

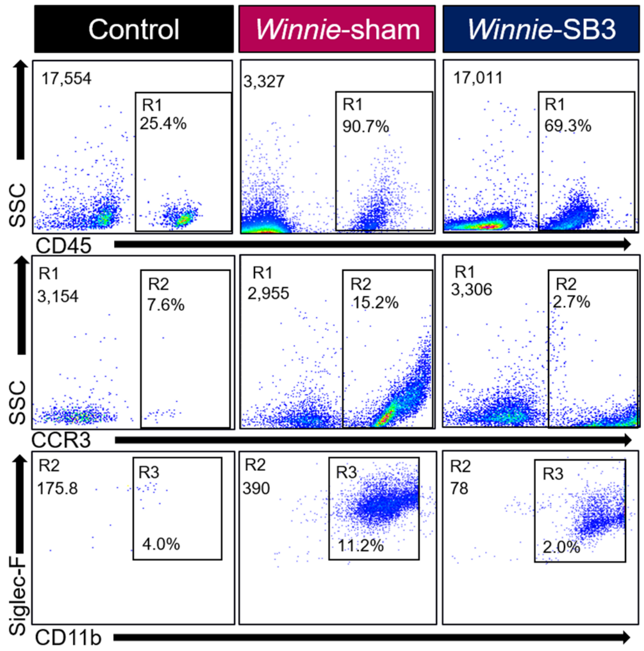

Figure S1: Eosinophil profiles were consistent with and without a marker for Siglec-F. (**Region (R) 1**) Viable cells were gated to isolate leukocytes based on SSC$^{LO/HI}$ against CD45^{+} cells. Subsequent gating was based on (**R2**) SSC^{HI} and CCR3^{+}, followed by (**R3**) CD11b^{+} and Siglec-F^{+} expressions to delineate the eosinophil subset.

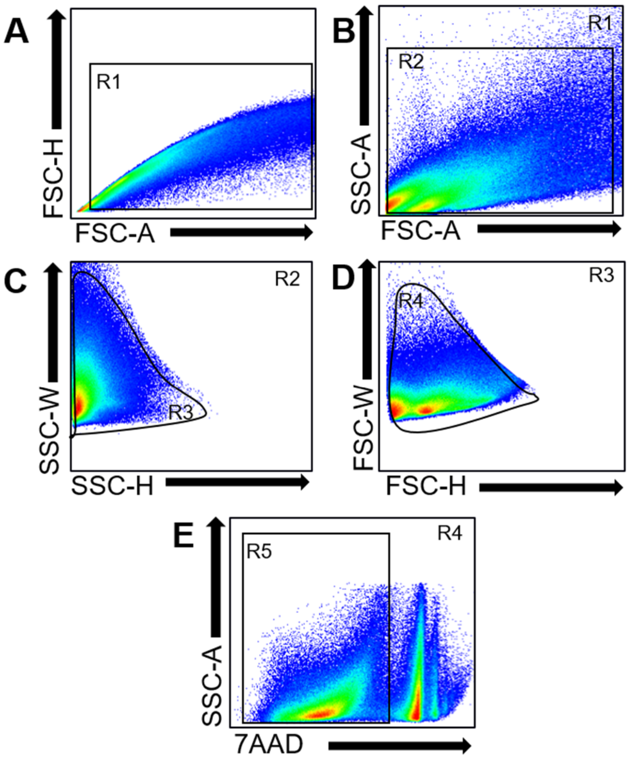

Figure S2: Application to discriminate all viable and live cells in biological specimen. (**Region (R) 1**) Cells were initially isolated based off FSC-H vs FSC-A profiles followed by (**R2**) SSC-A vs FSC-A cellular contours. Subsequent gating on excluding double discrimination based on (**R3**) SSC-W vs SSC-H and (**R4**) FSC-W vs FSC-H. (**R5**) Viable cells were discerned negatively expressed the 7AAD marker.
